# Prevalence and associated factors of suicidal ideation, plans, and attempts among LGBTQ+ individuals: A cross-sectional study in Vietnam

**DOI:** 10.1371/journal.pone.0357123

**Published:** 2026-08-28

**Authors:** Mai Tuyet Do, Tung Doan, Mai Pham, Binh Nguyen, Nhung Nguyen Do Hong, Kim-Duy Vu

**Affiliations:** 1 Vietnam National Cancer Hospital, Hanoi, Vietnam; 2 School of Preventive Medicine and Public Health, Hanoi Medical University, Hanoi, Vietnam; 3 Lighthouse Social Enterprise, Hanoi, Vietnam; 4 Universiteit Leiden, Leiden, Netherlands; 5 Thai Nguyen University of Education, Thai Nguyen, Viet Nam; 6 National Institute of Hygiene and Epidemiology, Hanoi, Vietnam; Australian Catholic University, AUSTRALIA

## Abstract

**Background:**

Suicidality is a critical public health concern among LGBTQ+ youth, yet evidence from Vietnam remains limited. This study estimated the prevalence of suicidal ideation, plans, and attempts and examined associated sociodemographic and psychological factors among Vietnamese LGBTQ+ youth.

**Methods:**

We conducted a community-based, online cross-sectional survey from February to April 2025. Participants included 384 LGBTQ+ individuals aged 18–24 years residing in Vietnam. Participants were recruited through the LGBTQ+ -led or LGBTQ+ -supportive social media and community networks. Suicidality was assessed alongside symptoms of depression, anxiety, and stress using the Depression Anxiety Stress Scales (DASS-21). Monthly income was self-reported and categorized into USD bands. Multivariable logistic regression models were employed to identify factors associated with lifetime suicidal outcomes.

**Results:**

The lifetime prevalence of suicidal ideation, plans, and attempts was 56.5%, 32.1%, and 16.9%, respectively. Recent (past 12 months) prevalence was 5.5%, 3.6%, and 3.1%. In multivariable analyses, stress [AOR = 4.52 (95% CI: 2.35–8.72)] and anxiety [AOR = 2.12 (95% CI: 1.03–4.34)] were associated with higher odds of suicidal ideation and plans. Anxiety emerged as the sole independent predictor of suicide attempts [AOR = 4.30 (95% CI: 1.28–14.41)]. Conversely, higher income (≥USD 386/month) and rural residence were associated with lower odds of suicidal ideation.

**Conclusions:**

Suicidality is highly prevalent among this accessible, predominantly urban, and connected subpopulation of LGBTQ+ youth in Vietnam. Anxiety and stress are the most robust proximal correlates of suicidal behaviors. Suicide prevention strategies for this population must incorporate routine screening for anxiety and stress, deliver LGBTQ + -affirming care, and address underlying economic vulnerabilities.

## Introduction

Studies across multiple settings have documented that suicidal ideation, suicide planning, and suicide attempts are serious public health concerns among lesbian, gay, bisexual, transgender, queer, and other sexual and gender minority (LGBTQ+) young people globally [1,2]. Evidence consistently shows that sexual and gender minority youth experience higher levels of psychological distress and suicide-related outcomes than their heterosexual and cisgender peers, reflecting the cumulative effects of stigma, discrimination, rejection, concealment, and limited access to affirming support [3,4].

LGBTQ+ populations face pervasive stigma, social rejection, and legal barriers to gender-affirming care, contributing to disproportionately high rates of depression, anxiety, suicidal ideation, and substance use disorders compared with the general population [5–7]. Minority Stress Theory posits that chronic exposure to prejudice, discrimination, and social adversity creates sustained psychological stress, leading to poorer mental health among LGBTQ+ individuals [8]. Minority Stress Theory provides the conceptual basis for interpreting suicide-related vulnerability among LGBTQ+ youth [5]. In this study, stigma, discrimination, concealment, rejection, and limited affirming services are understood as upstream minority-stress contexts. The variables available for analysis, including sociodemographic characteristics and DASS-21 depression, anxiety, and stress symptoms, should therefore be interpreted as downstream correlates rather than direct measures of minority stress mechanisms [5,9].

Accordingly, the analysis focuses on observable individual-level and structural factors that may reflect the downstream impacts of minority stress in shaping suicidality [10]. Empirical evidence from diverse settings supports this conceptual framework, demonstrating disproportionately high levels of suicidality among LGBTQ+ youth exposed to minority stressors. For example, The Trevor Project’s 2021 survey in U.S. found that 40% of LGBTQ+ youth had seriously considered suicide in the past year, with rates reaching 50% among transgender and gender-diverse youth [1]. Similarly, systematic reviews of contemporary data from developed countries consistently demonstrate a disproportionate burden of psychological distress, bullying, social isolation, and lack of family support among sexual minority youth, who remain significantly more likely to report symptoms of depression and suicidal ideation compared to heterosexual peers [2,11]. Transgender people face this risk nearly four times higher than cisgender people (people whose gender identity matches their sex assigned at birth [9]. Factors such as family rejection, bullying, and social isolation further exacerbate these risks, often resulting in long-term consequences including chronic depression and post-traumatic stress disorder (PTSD) [12,13].

In East and Southeast Asian contexts, LGBTQ+ individuals often encounter unique challenges stemming from traditional norms, collectivist values, and patriarchal expectations [14]. In Vietnam, societal stigma regarding gender and sexual diversity remains widespread, driving concealment and internalized negativity [15]. A 2023 study by Lighthouse Social Enterprise reported that 60% of LGBTQ+ respondents experienced discrimination, and over half exhibited symptoms of prolonged depression [16]. School-based violence is also prevalent, with over 70% of LGBTQ+ students reporting physical or verbal abuse [17]. LGBTQ+ youth also face major challenges in limited access to sexual and reproductive health services [18]. Furthermore, financial dependence on family and low income impede access to mental health and sexual health services [19,20]. Healthcare providers often lack the necessary training and sensitivity to serve this population effectively, with more than 60% reporting limited knowledge and nearly 72% indicating they had never provided care to LGBTQ+ patients [21].

Despite the increasing visibility of LGBTQ+ issues in Vietnam, there is a paucity of empirical data on the mental health challenges facing this population, particularly among youth who are often excluded from national surveillance systems. To address these gaps, this study aimed to examine the prevalence of lifetime suicidal ideation, plan, attempt, and associated factors among LGBTQ+ youth in Vietnam. Guided by Minority Stress Theory, this study focuses on how proximal psychological distress (depression, anxiety, stress) and distal structural factors (e.g., income, living context) are associated with suicidal outcomes (ideation, plans, and attempts). To our knowledge, this is among the first empirical analyses focusing specifically on suicide-related outcomes among LGBTQ+ young people in Vietnam. Findings from this study will contribute to a context-specific evidence base, informing the design of culturally appropriate interventions and policy advocacy to enhance the well-being of the LGBTQ+ community.

## Materials and methods

### Study design and procedure

We conducted an online cross-sectional survey using convenience sampling via social media and LGBTQ+ community networks. The Institutional Review Board approved the study protocol on February 7, 2025. Participant recruitment and data collection occurred between 26 February and 10 March, 2025; pre-approval activities were limited to internal questionnaire drafting and platform configuration, with no participant contact prior to IRB approval.

Participants were recruited through three principal channels: (i) the Pride MindZ Facebook page (~12,000 followers); (ii) the social media and direct messaging channels of Lighthouse Social Enterprise; and (iii) snowball outreach via partner LGBTQ+ community-based organizations. We acknowledge that this recruitment strategy enabled access to a sensitive and hard-to-reach population but may have introduced selection bias toward participants who were urban, internet-connected, more highly educated, and already connected to LGBTQ+ community networks.

Upon accessing the survey link, participants reviewed an information sheet detailing the study’s purpose, voluntary nature, and anonymity. Electronic informed consent was obtained from all participants before the survey began. The self-administered questionnaire took approximately 15–20 minutes to complete. To ensure data quality and prevent duplicate entries in the anonymous online format, a multi-step quality control protocol was implemented. At the platform level, the survey was configured to restrict submissions to one response per IP address and device. Additionally, attention-check items were embedded within the questionnaire to monitor respondent engagement. During data curation, submissions were systematically reviewed and excluded if they met any of the following criteria: duplicate IP addresses, inconsistent demographic responses, implausible completion times (e.g., completing the 15–20 minute survey in under 5 minutes), patterned response sets (e.g., straight-lining), or substantial missing data on primary outcome variables. To further encourage honest reporting on sensitive topics, no personally identifiable information was collected, and strict anonymity was maintained.

### Participants

Eligible participants were individuals aged 18–24 years residing in Vietnam (classified as youth in accordance with the United Nations definition of 15–24 years [22] and the Vietnam Youth Law 2020 which covers individuals aged 16–30 years [23]) who self-identified as LGBTQ+ (lesbian, gay, bisexual, transgender, queer, or other sexual/gender minorities). To estimate the required sample size, we conducted an a priori calculation using a single population proportion approach. Based on a lifetime suicidal ideation prevalence of 50.7% among LGBTQI+ people reported in a national survey in Thailand by Moallef et al. (2022) [24], a 95% confidence level, and an absolute precision of 5%, a minimum sample size of 384 was required. A total of 384 eligible individuals completed the survey and met the inclusion criteria, satisfying this requirement.

### Study measures

#### Independent variables.

Sociodemographic variables included age, sex assigned at birth (female reference), gender identity, sexual orientation, residence (urban/rural), migration status (resident in birthplace vs. migrated), education (<high school / high school / university / postgraduate), employment, relationship status (single / dating / married), cohabitation arrangement. Monthly income was self-reported as the participant's average income over the previous three months and categorized into approximate USD-equivalent bands based on the regional minimum wage standards and national average income benchmarks in Vietnam: < 193, 193- < 386, 386- < 578, 578- < 770, and>=770 USD per month [25,26].

#### Outcome variables.

The primary outcomes were lifetime suicidal ideation, suicide plans, and suicide attempts. Lifetime was defined as having ever experienced the outcome at any point in one's life. Self-harm was defined as intentional self-injurious behavior without stated suicidal intent, whereas suicide attempts were defined as self-injurious behaviors with the explicit intent to die. Suicidal ideation was assessed by asking whether participants had ever seriously thought about ending their life. Suicide planning was assessed by asking whether participants had ever made a specific plan to end their life. Suicide attempt was assessed by asking whether participants had ever actively attempted to end their life. For descriptive purposes, participants who reported each outcome were further asked about timing, categorized as more than two years ago, within the past two years, within the past 12 months, or within the past three months. These recent categories were mutually exclusive descriptive categories and were not used as the primary regression outcomes because event counts were small. Exact item wording is provided in the S1 Supplementary.

While we acknowledge that a validated multi-item assessment would offer greater psychometric precision, the use of a single-item measure was considered appropriate given that the primary aim of this study was prevalence estimation rather than clinical assessment. The single-item measure also is widely supported by established public health surveillance systems, such as the CDC’s Youth Risk Behavior Surveillance System (YRBSS) or the Trevor Project National Survey [1,27]. Additionally, this approach helps minimize participant distress and survey fatigue, and reduce dropout rates, which is a critical consideration in online research on sensitive topics.

#### Psychological distress.

Symptoms of depression, anxiety, and stress over the past week were measured using the Vietnamese version of the 21-item Depression Anxiety Stress Scales (DASS-21) [28]. Participants rated symptom severity over the past week on a 4-point Likert scale (0 = “Did not apply to me at all” to 3 = “Applied to me very much”). The Vietnamese DASS-21 has been validated in Vietnamese adolescents [29], demonstrating good reliability and a stable factor structure in this age group, which closely aligns with the developmental stage of our study population. While dedicated validation in LGBTQ+ Vietnamese youth has not yet been conducted, the strong internal consistency observed in our sample (Cronbach's α > 0.90 for all three subscales). In this study, it was used as a symptom screening tool rather than a diagnostic instrument.

### Statistical analysis

Descriptive statistics (frequencies, percentages, means, and standard deviations) summarized demographics and mental health indicators. Prevalence rates were reported with 95% confidence intervals (CI). Bivariate analyses (chi-square, Fisher’s exact tests, t-tests) were used to screen for associations. Variables were selected using the Purposeful Selection approach of Hosmer, Lemeshow [30]. Candidate variables with univariable Wald p < 0.20 were entered into the initial multivariable model, alongside five pre-specified forced variables: DASS-21 anxiety, stress, depression, mean-centered age, and monthly income. Variables with multivariable p > 0.10 were considered for removal but were retained if their exclusion changed any remaining coefficient by ≥ 20% (indicating confounding). Variables excluded at the univariable stage were then re-evaluated and re-added if p < 0.10. Multicollinearity was assessed via variance inflation factor (VIF), with VIF ≥ 5 considered moderate and ≥ 10 severe. Model goodness-of-fit was evaluated using the Hosmer–Lemeshow test and Nagelkerke pseudo-R^2^. Model robustness was assessed using the events-per-variable (EPV) ratio, with an EPV ≥ 10 considered optimal. All analyses were conducted using Stata 18.0 software, with a two-tailed p-value of less than 0.05 regarded as statistically significant.

### Ethical considerations

The study protocol was reviewed and approved by the Institutional Review Board of the Hanoi University of Public Health (Decision No. 29/2025/YTCC-HD3, dated February 7, 2025). The study was conducted in accordance with the Declaration of Helsinki. Prior to data collection, all participants were provided with detailed information about the study's purpose, confidentiality, and their rights. Participants provided electronic informed consent by clicking an “I Agree” button before accessing the survey questions. Participation was entirely voluntary, and respondents could withdraw from the survey at any time without consequence. Strict confidentiality and anonymity were maintained throughout the data collection and analysis process. Before proceeding to subsequent sections, participants were asked to answer a check-in question to confirm their willingness and ability to continue with the questionnaire. Because the questionnaire included sensitive questions on self-harm and suicide-related experiences, participants were provided with information on LGBTQ + -affirming mental health support and referral options. Participants who reported distress or suicide-related concerns during study participation were encouraged to seek support from qualified professionals and community-based support services. Participants reporting distress were provided with information on mental health support services and crisis hotlines. No adverse events were reported during the data collection period.

## Results

### Demographic characteristics

As shown in Table 1, the study included 384 participants with a mean age of 21.3 years (SD = 1.8). The vast majority resided in urban areas (94.5%) and had a history of migration (66.9%). Regarding education, 71.9% held a university or college degree. In terms of relationships, 65.1% were single, while 33.3% were dating. More than half of the participants lived with family (53.9%). Financially, 53.4% reported a monthly income of less than USD 193, and 18.0% reported insufficient funds for basic expenses. The sample was predominantly assigned female at birth (60.9%). Regarding gender identity, 44.8% identified as cisgender women, 31.8% as cisgender men, and 18.8% as non-binary. Sexual orientation was primarily gay/lesbian (54.7%) or bisexual (29.4%).The sample was predominantly urban, university/college educated, and assigned female at birth. These characteristics should be interpreted as reflecting an online-recruited accessible sample rather than population-level estimates for all LGBTQ+ youth in Vietnam.

**Table 1:** General demographic characteristics (N = 384).

Demographic characteristics		n	%
Living area	Urban	363	94.53
	Rural	21	5.47
Migration status	No	127	33.07
	Yes	257	66.93
Education level	<High school	10	2.60
	High school	84	21.88
	University/College	276	71.88
	Postgraduate	14	3.65
Current relationship	Single	250	65.10
	Dating	128	33.33
	Married	6	1.56
Cohabitation status	Living alone	99	25.78
	Living with family	207	53.91
	Living with a partner	13	3.39
	Living with a LGBTQ+ friend (not partner)	30	7.81
	Living with a non-LGBTQ+ friend	35	9.11
Current job	Studying, not working yet	210	54.69
	Employed	64	16.67
	Self-employed	96	25.00
	No job	4	1.04
	Other^a^	10	2.60
Average income for the last 3 months (equivalent USD/month)	< 193	205	53.39
	193- < 386	91	23.70
	386- < 578	64	16.67
	578- < 770	17	4.43
	≥ 770	7	1.82
Financial capacity	Insufficient basic expenses	69	17.97
	Enough basic expenses, no surplus	203	52.86
	Enough expenses and surplus	112	29.17
Sex assigned at birth	Female	234	60.94
	Male	150	39.06
Gender identity	Cisgender man	122	31.77
	Cisgender woman	172	44.79
	Transgender man	12	3.12
	Transgender woman	6	1.56
	Non-binary	72	18.75
Sexual orientation	Gay/Lesbian	210	54.69
	Heterosexual	6	1.56
	Bisexual	113	29.43
	Asexual	11	2.86
	Pansexual	9	2.34
	Demisexual	3	0.78
	Unknown^c^	32	8.33

Note: ^b^”Other” job category includes freelancers, part-time seasonal workers, and individuals currently unable to work due to medical or personal reasons; ^c^"Unknown” sexual orientation category includes individuals who identified as questioning, those who preferred not to label their sexual orientation (unlabeled), or those who chose not to disclose this information.

### Prevalence of suicidality

Lifetime prevalence was 56.5% (95% CI: 51.5–61.5; n = 217) for suicidal ideation, 32.0% (95% CI: 27.4–37.0; n = 123) for suicide plans, and 16.9% (95% CI: 13.4–21.0; n = 65) for suicide attempts. Recency categories were mutually exclusive, with the “past 12 months” category excluding the “past 3 months” category. Past-12-months-but-not-past-3-months prevalences were 5.5% (n = 21) for ideation, 7.8% (n = 30) for plans, and 3.1% (n = 12) for attempts. Past-3-month prevalences were 0% for ideation, 1.6% (n = 6) for plans, and 0.5% (n = 2) for attempts (Table 2).

**Table 2:** Prevalence of lifetime suicidal ideation, plan, and attempts (N = 384).

Expression	Never n (%)	Ever had n (%)			
		More than 2 years ago	Over the past 2 years	Within the past 12 months	Within the past 3 months
Suicidal attempt	319 (83.07)	45 (11.7)	6 (1.6)	12 (3.1)	2 (0.5)
Suicidal ideations	167 (43.5)	134 (34.9)	62 (16.1)	21 (5.5)	0
Suicidal plans	261 (67.9)	65 (16.9)	22 (5.7)	30 (3.6)	6 (1.6)

As stated in the manuscript, the exact item wordings above included highly granular timeframes to capture acute clinical risk. For the descriptive analysis presented in Table 2, these responses were coded into mutually exclusive categories based on the most recent occurrence. Specifically, granular responses such as “within the past week,” “within the past 3 days,” or “currently” were collapsed into the broader “Within the past 3 months” category. Similarly, “within the past 6 months” was collapsed into the “Within the past 12 months” category. This coding strategy ensured mutually exclusive descriptive categories while addressing the small event counts in the highly granular timeframes.

### Factors associated with suicidal ideation

In univariable analyses, all three DASS-21 subscales were strongly associated with suicidal ideation, including anxiety [COR = 5.24 (95% CI: 3.00–9.17)], stress [COR = 6.00 (95%CI: 3.73–9.65)], and depression [COR = 4.18 (95%CI: 2.55–6.85)]. Among sociodemographic variables, rural residence [COR = 0.36 (95%CI: 0.14–0.92)], migration [COR = 0.61 (95%CI:0.39–0.94)], employment [COR = 0.64 (95%CI: 0.42–0.96)], higher monthly income [COR = 0.47 (95%CI: 0.29–0.76)], and male sex assigned at birth [COR = 0.38 (95%CI: 0.25–0.57)] were associated with lower crude odds (Table 3). In the multivariable model, stress [AOR = 4.61 (95% CI: 2.39–8.86)] was the strongest psychological correlate; anxiety [AOR = 1.94 (95%CI: 0.94–3.99)] showed a positive but non-significant association; depression [AOR = 1.40 (95%CI: 0.68–2.88)] was attenuated. Sociodemographic correlates included rural residence [AOR = 0.24 (95%CI: 0.08–0.70)], higher monthly income [AOR = 0.45 (95%CI: 0.24–0.84)], male sex assigned at birth [AOR = 0.42 (95%CI: 0.26–0.69)], and dating relationship vs. single [AOR = 2.00 (95%CI: 1.17–3.43)]. Notably, while rural residence appeared associated with lower odds of suicidal ideation, this subgroup was very small (n = 21, 5.5%); therefore, this estimate is statistically unstable and should be interpreted with extreme caution. The model fit well (Nagelkerke R² = 0.336; Hosmer–Lemeshow p = 0.602).

**Table 3:** Logistic regression analysis of factors associated with suicidal ideation among study participants.

Characteristics	Outcome		Univariable		Multivariable	
	Yes (N = 217)	No (N = 167)	COR	95% CI	AOR	95% CI
**Anxiety**						
No	20 (25.6%)	58 (74.4%)	ref		ref	
Yes	197 (64.4%)	109 (35.6%)	5.24***	[3.00,9.17]	1.94	[0.94,3.99]
**Stress**						
No	34 (27.9%)	88 (72.1%)	ref		ref	
Yes	183 (69.8%)	79 (30.2%)	6.00***	[3.73,9.65]	4.61***	[2.39,8.86]
**Depression**						
No	30 (30.9%)	67 (69.1%)	ref		ref	
Yes	187 (65.2%)	100 (34.8%)	4.18***	[2.55,6.85]	1.40	[0.68,2.88]
**Age**						
Mean (SD)	21.25 (1.76)	21.35 (1.87)	0.97	[0.87,1.09]	1.11	[0.95,1.31]
**Living area**						
Urban	210 (57.9%)	153 (42.1%)	ref		ref	
Rural	7 (33.3%)	14 (66.7%)	0.36*	[0.14,0.92]	0.24**	[0.08,0.70]
**Migration status**						
No	82 (64.6%)	45 (35.4%)	ref		ref	
Yes	135 (52.5%)	122 (47.5%)	0.61*	[0.39,0.94]	0.65	[0.38,1.11]
**Education level**						
<High school	5 (50.0%)	5 (50.0%)	0.76	[0.22,2.68]	—	
High school	44 (52.4%)	40 (47.6%)	0.81	[0.50,1.31]	—	
Graduate	163 (59.1%)	113 (40.9%)	1.44	[0.92,2.26]	—	
Postgraduate	5 (35.7%)	9 (64.3%)	ref		ref	
**Current relationship**						
Single	133 (53.2%)	117 (46.8%)	ref		ref	
Dating	80 (62.5%)	48 (37.5%)	1.45	[0.94,2.23]	2.00*	[1.17,3.43]
Married	4 (66.7%)	2 (33.3%)	1.55	[0.28,8.56]	—	
**Cohabitation status**						
Living alone	47 (47.5%)	52 (52.5%)	ref		ref	
Living with family	123 (59.4%)	84 (40.6%)	1.29	[0.86,1.94]	—	
Living with partner	6 (46.2%)	7 (53.8%)	0.65	[0.21,1.97]	0.31	[0.09,1.16]
Living with LGBTQ+ friend	19 (63.3%)	11 (36.7%)	1.36	[0.63,2.94]	—	
Living with non-LGBTQ+ friend	22 (62.9%)	13 (37.1%)	1.34	[0.65,2.74]	—	
**Current job**						
Unemployed/Studying	137 (61.2%)	87 (38.8%)	ref		ref	
Employed	80 (50.0%)	80 (50.0%)	0.64*	[0.42,0.96]	0.87	[0.46,1.65]
**Average income (USD/month)**						
< 386	180 (60.8%)	116 (39.2%)	ref		ref	
≥ 386	37 (42.0%)	51 (58.0%)	0.47**	[0.29,0.76]	0.45*	[0.24,0.84]
**Financial capacity**						
Not enough	46 (66.7%)	23 (33.3%)	ref		ref	
Enough	171 (54.3%)	144 (45.7%)	0.59	[0.34,1.03]	—	
**Sex assigned at birth**						
Female	154 (65.8%)	80 (34.2%)	ref		ref	
Male	63 (42.0%)	87 (58.0%)	0.38***	[0.25,0.57]	0.42***	[0.26,0.69]

COR = crude odds ratio; AOR = adjusted odds ratio; 95% CI = 95% confidence interval. * p < 0.05; ** p < 0.01; *** p < 0.001. Multivariable model selected via Purposeful Selection (Hosmer-Lemeshow-Sturdivant 2013); AOR is reported only for variables retained in the final model. “—” indicates the variable was not retained. Continuous age was mean-centered (mean = 21.3 years). Model diagnostics: Nagelkerke R^2^ = 0.336; Hosmer–Lemeshow χ^2^ = 6.41 (p = 0.602); maximum VIF = 7.23.

### Factors associated with suicidal plans

In univariable analyses, all three DASS-21 subscales were significantly associated with suicide plans (anxiety COR 5.27; stress COR 4.20; depression COR 3.30; all p < 0.001). No sociodemographic variable met the Step 1 univariable screening threshold of p < 0.20 (Table 4). In the multivariable model (containing only the five forced variables), both anxiety [AOR = 2.78 (95%CI: 1.16–6.68)] and stress [AOR = 2.74 (95%CI: 1.35–5.56)] remained independently associated with suicide plans; depression [AOR = 1.17 (95%CI: 0.54–2.53)] was attenuated. The model fit well (Nagelkerke R^2^ = 0.137; Hosmer–Lemeshow p = 0.238).

**Table 4:** Logistic regression analysis of factors associated with suicidal plans among study participants.

Characteristics	Outcome		Univariable		Multivariable	
**Yes (N = 123)**	**No (N = 261)**	**COR**	**95% CI**	**AOR**	**95% CI**
**Anxiety**						
No	8 (10.3%)	70 (89.7%)	ref		ref	
Yes	115 (37.6%)	191 (62.4%)	5.27***	[2.45,11.35]	2.78*	[1.16,6.68]
**Stress**						
No	17 (13.9%)	105 (86.1%)	ref		ref	
Yes	106 (40.5%)	156 (59.5%)	4.20***	[2.38,7.41]	2.74**	[1.35,5.56]
**Depression**						
No	15 (15.5%)	82 (84.5%)	ref		ref	
Yes	108 (37.6%)	179 (62.4%)	3.30***	[1.81,6.01]	1.17	[0.54,2.53]
**Age**						
Mean (SD)	21.47 (1.82)	21.21 (1.80)	1.08	[0.96,1.22]	1.12	[0.98,1.29]
**Living area**						
Urban	117 (32.2%)	246 (67.8%)	ref		ref	
Rural	6 (28.6%)	15 (71.4%)	0.84	[0.32,2.22]	—	
**Migration status**						
No	36 (28.3%)	91 (71.7%)	ref		ref	
Yes	87 (33.9%)	170 (66.1%)	1.29	[0.81,2.06]	—	
**Education level**						
<High school	2 (20.0%)	8 (80.0%)	0.52	[0.11,2.50]	—	
High school	26 (31.0%)	58 (69.0%)	0.94	[0.56,1.58]	—	
Graduate	93 (33.7%)	183 (66.3%)	1.32	[0.81,2.16]	—	
Postgraduate	2 (14.3%)	12 (85.7%)	ref		ref	
**Current relationship**						
Single	76 (30.4%)	174 (69.6%)	ref		ref	
Dating	45 (35.2%)	83 (64.8%)	1.24	[0.79,1.94]	—	
Married	2 (33.3%)	4 (66.7%)	1.06	[0.19,5.88]	—	
**Cohabitation status**						
Living alone	25 (25.3%)	74 (74.7%)	ref		ref	
Living with family	73 (35.3%)	134 (64.7%)	1.38	[0.90,2.14]	—	
Living with partner	3 (23.1%)	10 (76.9%)	0.63	[0.17,2.32]	—	
Living with LGBTQ+ friend	12 (40.0%)	18 (60.0%)	1.46	[0.68,3.13]	—	
Living with non-LGBTQ+ friend	10 (28.6%)	25 (71.4%)	0.84	[0.39,1.80]	—	
**Current job**						
Unemployed/Studying	68 (30.4%)	156 (69.6%)	ref		ref	
Employed	55 (34.4%)	105 (65.6%)	1.20	[0.78,1.85]	—	
**Average income (USD/month)**						
< 386	95 (32.1%)	201 (67.9%)	ref		ref	
≥ 386	28 (31.8%)	60 (68.2%)	0.99	[0.59,1.65]	0.87	[0.50,1.53]
**Financial capacity**						
Not enough	20 (29.0%)	49 (71.0%)	ref		ref	
Enough	103 (32.7%)	212 (67.3%)	1.19	[0.67,2.11]	—	
**Sex assigned at birth**						
Female	78 (33.3%)	156 (66.7%)	ref		ref	
Male	45 (30.0%)	105 (70.0%)	0.86	[0.55,1.33]	—	

COR = crude odds ratio; AOR = adjusted odds ratio. * p < 0.05; ** p < 0.01; *** p < 0.001. Multivariable model retained only the five forced predictors; live_family was tested at Step 4 and removed (Δ = 16.6% < 20% threshold). Model diagnostics: Nagelkerke R^2^ = 0.137; Hosmer–Lemeshow χ^2^ = 10.41 (p = 0.238); maximum VIF = 7.10.

### Factors associated with suicide attempts

In univariable analyses, anxiety [COR = 4.61 (95%CI: 1.62–13.09)], dating relationship [COR = 1.93 (95%CI: 1.12–3.33)], living with a partner [COR = 3.24 (95%CI: 1.02–10.24)], and living with an LGBTQ+ friend [COR = 2.72 (95%CI:1.21–6.12)] were significantly associated with suicide attempts. Stress and depression did not reach significance at the univariable level (Table 5). In the multivariable model, anxiety [AOR = 4.01 (95%CI: 1.23–13.13)] was the principal independent predictor of suicide attempts. Living with an LGBTQ+ friend [AOR = 2.36 (95%CI: 1.00–5.57)] reached borderline significance. Dating, living with a partner, and being employed were retained as confounders of the age coefficient (Step 4: ≥ 20% coefficient change when removed) but did not themselves reach significance. The model fit well (Nagelkerke R^2^ = 0.109; Hosmer–Lemeshow p = 0.393).

**Table 5:** Logistic regression analysis of factors associated with suicidal attempts among study participants.

Characteristics	Outcome		Univariable		Multivariable	
	Yes (N = 65)	No (N = 319)	COR	95% CI	AOR	95% CI
**Anxiety**						
No	4 (5.1%)	74 (94.9%)	ref		ref	
Yes	61 (19.9%)	245 (80.1%)	4.61**	[1.62,13.09]	4.01*	[1.23,13.13]
**Stress**						
No	14 (11.5%)	108 (88.5%)	ref		ref	
Yes	51 (19.5%)	211 (80.5%)	1.86	[0.99,3.52]	1.47	[0.65,3.32]
**Depression**						
No	12 (12.4%)	85 (87.6%)	ref		ref	
Yes	53 (18.5%)	234 (81.5%)	1.60	[0.82,3.15]	0.76	[0.31,1.87]
**Age**						
Mean (SD)	21.55 (1.90)	21.24 (1.79)	1.10	[0.95,1.28]	1.01	[0.84,1.22]
**Living area**						
Urban	63 (17.4%)	300 (82.6%)	ref		ref	
Rural	2 (9.5%)	19 (90.5%)	0.50	[0.11,2.21]	—	
**Migration status**						
No	17 (13.4%)	110 (86.6%)	ref		ref	
Yes	48 (18.7%)	209 (81.3%)	1.49	[0.82,2.71]	—	
**Education level**						
<High school	2 (20.0%)	8 (80.0%)	1.23	[0.26,5.95]	—	
High school	13 (15.5%)	71 (84.5%)	0.87	[0.45,1.69]	—	
Graduate	49 (17.8%)	227 (82.2%)	1.24	[0.67,2.29]	—	
Postgraduate	1 (7.1%)	13 (92.9%)	ref		ref	
**Current relationship**						
Single	34 (13.6%)	216 (86.4%)	ref		ref	
Dating	30 (23.4%)	98 (76.6%)	1.93*	[1.12,3.33]	1.54	[0.86,2.76]
Married	1 (16.7%)	5 (83.3%)	0.98	[0.11,8.54]	—	
**Cohabitation status**						
Living alone	16 (16.2%)	83 (83.8%)	ref		ref	
Living with family	32 (15.5%)	175 (84.5%)	0.80	[0.47,1.36]	—	
Living with partner	5 (38.5%)	8 (61.5%)	3.24*	[1.02,10.24]	2.20	[0.66,7.35]
Living with LGBTQ+ friend	10 (33.3%)	20 (66.7%)	2.72*	[1.21,6.12]	2.36*	[1.00,5.57]
Living with non-LGBTQ+ friend	2 (5.7%)	33 (94.3%)	0.28	[0.06,1.18]	—	
**Current job**						
Unemployed/Studying	31 (13.8%)	193 (86.2%)	ref		ref	
Employed	34 (21.2%)	126 (78.8%)	1.68	[0.98,2.87]	1.42	[0.69,2.90]
**Average income (USD/month)**						
< 386	45 (15.2%)	251 (84.8%)	ref		ref	
≥ 386	20 (22.7%)	68 (77.3%)	1.64	[0.91,2.96]	1.24	[0.62,2.48]
**Financial capacity**						
Not enough	10 (14.5%)	59 (85.5%)	ref		ref	
Enough	55 (17.5%)	260 (82.5%)	1.25	[0.60,2.59]	—	
**Sex assigned at birth**						
Female	37 (15.8%)	197 (84.2%)	ref		ref	
Male	28 (18.7%)	122 (81.3%)	1.22	[0.71,2.10]	—	

COR = crude odds ratio; AOR = adjusted odds ratio. * p < 0.05; ** p < 0.01; *** p < 0.001. Multivariable model retained nine predictors; dating, live_partner, and employed were retained as confounders of age (Step 4 coefficient change > 20%). Model diagnostics: Nagelkerke R^2^ = 0.109; Hosmer–Lemeshow χ^2^ = 8.43 (p = 0.393); maximum VIF = 7.12.

## Discussion

In this community-based study of LGBTQ+ young adults in Vietnam, we observed a substantial burden of mental health symptoms and suicidality. A significant proportion of participants reported clinically meaningful symptoms on the DASS-21, with anxiety and stress being particularly prominent. These findings align with previous studies in Asian LGBTQ+ samples documenting high prevalence of mental disorders [31] and reports from Vietnam documenting widespread discrimination and prolonged depressive symptoms [32].

The prevalence of suicidal phenomena in our sample – spanning ideation, planning, and attempts – is comparable to global data indicating elevated risks among LGBTQ+ youth. Reviews have similarly highlighted elevated risks of depression, anxiety, suicidality, and related health harms among LGBTQ+ youth, reinforcing the public health significance of mental health disparities in sexual and gender minority populations [2,33]. The previous large-scale reports indicated high rates of suicidal thoughts and self-harm among LGBTQ+ youth [1], and studies among LGBTQ+ youth in Quebec showing elevated mental health symptoms and suicide-related outcomes [34]. Importantly, the presence of recent and current suicidal phenomena suggests not only historical vulnerability but also ongoing risk. Given that a history of suicidal ideation or behavior is a strong predictor of future suicidal outcomes, these findings indicate an urgent need for early detection, crisis response capacity, and sustained follow-up support for LGBTQ+ youth in Vietnam.

Our findings can be understood through psychosocial frameworks, particularly stress accumulation and socio-environmental pathways. Minority Stress Theory posits that exposure to stigma, expectations of rejection, concealment, and internalized stigma generate chronic psychological strain and elevate psychiatric risk among sexual and gender minorities [8]. In addition, structural stigma such as discriminatory norms, limited legal protections, and hostile social environments has been associated with increased suicide risk among LGBTQ+ youth [35]. In Vietnam and similar East Asian contexts, traditional gender norms and strong family expectations may intensify these stress processes, especially for young people who remain financially dependent and socially constrained. This sociocultural environment may help explain the elevated levels of anxiety and stress observed in our sample, and may also contribute to both the persistence of suicidal outcomes and barriers to accessing affirming care. These findings are consistent with Minority Stress Theory, suggesting that prolonged exposure to stressors may be reflected in heightened anxiety and stress, which in turn are associated with suicidal outcomes.

Anxiety and stress emerged as the most consistent correlates across all outcomes. Both were independently associated with suicidal ideation and suicide planning in multivariable models, and anxiety remained strongly associated with suicide attempts. This pattern is clinically meaningful, suggesting that anxiety-related arousal and sustained stress exposure are strongly associated with suicidal thinking and planning, particularly in contexts of chronic minority stress [8,35]. Given evidence that LGBTQ+ youth face elevated risks of psychiatric symptoms and suicidality with downstream health impacts [33], our findings reinforce the importance of routine assessment of anxiety and stress – not only depressive symptoms – in suicide risk screening and prevention approaches for LGBTQ+ young people.

In this sample, depression was associated with suicidal ideation and planning in unadjusted analyses but was attenuated after adjustment. This attenuation may reflect overlap among symptom domains, such that anxiety and stress capture more proximal distress at the time of assessment, or potential collinearity that reduces the independent contribution of depressive symptoms in multivariable models. The attenuation of depression may partly reflect temporal mismatch, as DASS-21 assesses symptoms over the past week, whereas suicidality outcomes were measured over the lifetime. Regardless of mechanism, the results suggest that screening strategies focusing only on depression may miss individuals with clinically significant suicide risk driven primarily by anxiety and stress. This has practical implications for intervention selection, particularly for brief, scalable strategies that target acute distress and strengthen coping and safety planning, while maintaining capacity to address comorbidity.

Several social and structural correlates also appear relevant to suicidal ideation. Higher monthly income remained protective after adjustment, suggesting that economic security may buffer suicide risk by reducing material hardship and improving access to supportive resources. This aligns with prior evidence that LGBTQ+ youth often experience financial barriers to care, particularly when dependent on family resources [19], and that stigma-related barriers remain common in mental health help-seeking [18,36]. Considering that many participants reported low income and limited financial capacity, economic vulnerability may represent a modifiable target for prevention through low-cost services, linkage to social support programs, and community-based, youth-friendly interventions.

Unexpectedly, rural residence was associated with lower odds of suicidal ideation compared with urban residence after adjustment. The apparent protective association for rural residence should be interpreted with particular caution because rural participants represented only a small proportion of the sample, leading to wide confidence intervals. While this could theoretically reflect differences in urban competitive education or employment pressures and online minority stressors, this estimate may be unstable and should be considered exploratory until replicated in studies with geographically balanced sampling and sufficient rural LGBTQ+ participants. Further qualitative research and longitudinal designs are warranted to clarify whether rural residence is truly protective or whether the observed association reflects unmeasured confounding and measurement factors.

Relationship status was significantly associated with suicidal ideation, with individuals in a dating relationship showing higher odds compared with those who were single. While intimate relationships can provide social support, they may also introduce additional stressors in stigmatizing contexts such as conflict, concealment pressures, and fear of rejection particularly where LGBTQ+ relationships remain socially stigmatized and lack structural protection [8]. This pattern likely reflects relational stress and concealment demands rather than a direct causal effect. It also underscores the need for relationship-informed mental health support, including psychoeducation on healthy relationships, conflict management, and safety planning, alongside broader efforts to reduce stigma and foster more affirming interpersonal environments [35].

Beyond individual risk factors, our findings should be interpreted against the broader context of Minority Stress Theory. While upstream constructs such as family rejection, school violence, and concealment were not directly measured in our survey, existing literature suggests they are critical drivers of the downstream psychological distress (anxiety and stress) observed in our sample [12,13,17]. Because these variables were unmeasured, they serve as plausible contextual explanations rather than empirically tested predictors in the current study.

### Implications

These findings carry actionable implications for service delivery. First, suicide prevention protocols for LGBTQ+ youth must integrate routine screening for anxiety and stress, rather than relying solely on depression assessments. Second, given that higher income was protective against suicidal ideation, structural barriers to care—particularly financial constraints—must be addressed through low-threshold, affordable referral pathways. Finally, strengthening provider competencies to deliver LGBTQ + -affirming care is critical to mitigating the fear of discrimination in healthcare settings [21,37].

### Strengths and limitations

This online, community-recruited quantitative assessment provides exploratory evidence on suicide-related outcomes and associated factors among an accessible sample of LGBTQ+ youth in Vietnam, generating timely evidence for an under-researched population. The anonymous online design may have facilitated disclosure of sensitive experiences such as self-harm and suicidal thoughts, while the inclusion of diverse sexual orientations and gender identities allowed for an initial characterization of heterogeneity within LGBTQ+ subgroups. In addition, the study examined both symptom domains (depression, anxiety, stress) and suicide-related outcomes across a spectrum (self-harm, ideation, plans, and attempts), supporting a more clinically actionable understanding of risk.

This study has several limitations that should be considered when interpreting the findings. First, the use of online convenience sampling introduces selection bias, resulting in a sample that is predominantly urban, socially connected, and relatively well-educated. As such, the findings may not be generalized to the broader or more marginalized LGBTQ+ populations in Vietnam, particularly those living in rural areas, those with lower educational attainment, or those lacking digital connectivity, who may face different or more severe vulnerabilities. Second, all measures were based on self-report, which may be subject to recall bias and social desirability bias, particularly for sensitive topics such as suicidality. Third, the assessment of suicidality relied on single-item self-report measures rather than comprehensive clinical interviews or multi-item validated scales, which may affect psychometric precision, and there is a temporal mismatch between the measurement of psychological distress and suicidal outcomes. While DASS-21 assesses symptoms over the past week, suicidality was measured using lifetime indicators, which may affect the interpretation of associations. The DASS-21 subscales are conceptually overlapping, this shared variance, combined with the temporal mismatch, likely explains why depression attenuated in multivariable models after adjusting for anxiety and stress. Furthermore, while the DASS-21 showed high internal consistency in our sample, it has not been specifically validated for LGBTQ+ youth in Vietnam; future identity-specific psychometric evaluation is warranted. Fourth, the cross-sectional design precludes causal inference. The observed associations should be interpreted as correlational rather than causal relationships. Some subgroup analyses (e.g., rural participants) were based on small sample sizes, which may lead to unstable estimates and should be interpreted with caution. Finally, important upstream minority stressors, such as experiences of stigma, trauma, family rejection, and social support, were not measured in this study. The observed associations should be interpreted as correlational, and future research should incorporate these variables to provide a more comprehensive model of suicide risk.

### Future research

Longitudinal studies are needed to establish temporal relationships and test whether anxiety and stress prospectively predict progression from ideation to planning and attempts. More representative sampling approaches (e.g., probability or respondent-driven sampling) and oversampling of rural and transgender/nonbinary subgroups would strengthen generalizability. Mixed-methods work could clarify mechanisms related to family dynamics, school violence, concealment, and barriers to care, and help interpret unexpected associations. Future studies should also validate suicidality/self-harm measures for Vietnamese LGBTQ+ populations and include key constructs such as minority stress, stigma, social support, and service quality. Finally, intervention trials should evaluate scalable LGBTQ + -affirming, stepped-care models that explicitly target anxiety and stress and improve access to affordable, competent services.

## Conclusion

In summary, suicidal expressions were common among young LGBTQ+ individuals in Vietnam, including recent and current self-harm and suicidal ideation. Anxiety and stress were the most robust correlates across suicidal outcomes, while higher income appeared protective for suicidal ideation. These findings support the need for comprehensive, LGBTQ + -affirming suicide prevention efforts that integrate anxiety/stress assessment, timely psychological support, and attention to socioeconomic vulnerabilities.

## Supporting information

S1 Supplementary Suicidality and Self-Harm Assessment Questionnaire. (DOCX)
