# Supplementary material for: Prevalence and associated factors of suicidal ideation, plans, and attempts among LGBTQ+ individuals: A cross-sectional study in Vietnam
Source: PLoS One. 2026 Aug 28;21(8):e0357123. doi: 10.1371/journal.pone.0357123 (PMC13524257; doi:10.1371/journal.pone.0357123)
Supplement: Supplementary file 1 [file pone.0357123.s001.docx]

**Supplementary S1. Suicidality and Self-Harm Assessment Questionnaire**

*(General Instruction for participants: Please read carefully and select the option that represents your* ***most recent*** *experience).*

**B1. Have you ever intentionally harmed yourself without suicidal intent?**

*[Note for reviewers/readers: This item was included in the survey to differentiate non-suicidal self-injury from actual suicide attempts. As the current manuscript focuses specifically on suicidality (ideation, plans, and attempts), data from this item are not reported in the main results].*

- Never
- Yes, more than 2 years ago
- Yes, within the past 2 years
- Yes, within the past 12 months
- Yes, within the past 3 months
- Yes, currently

**B2. Have you ever seriously thought about ending your life?**

- Never → *Skip to C1*
- Yes, more than 2 years ago → *Go to B4*
- Yes, within the past 2 years → *Go to B4*
- Yes, within the past 12 months → *Go to B4*
- Yes, within the past 3 months → *Go to B3*
- Yes, currently → *Go to B3*

**B3. During the past 3 months, how often have you had thoughts about ending your life?**

- Never
- Rarely
- Sometimes
- Often
- Always

**B4. Have you ever made a specific plan to end your life?**

- Never
- Yes, more than 2 years ago
- Yes, within the past 2 years
- Yes, within the past 12 months
- Yes, within the past 3 months
- Yes, within the past week

**B5. Have you ever actively attempted to end your life?**

- Never
- Yes, more than 2 years ago
- Yes, within the past 2 years
- Yes, within the past year
- Yes, within the past 6 months
- Yes, within the past 3 months
- Yes, within the past month
- Yes, within the past week
- Yes, within the past 3 days
